# The Major Storage Protein in Potato Tuber Is Mobilized by a Mechanism Dependent on Its Phosphorylation Status

**DOI:** 10.3390/ijms20081889

**Published:** 2019-04-17

**Authors:** Javier Bernal, Daniel Mouzo, María López-Pedrouso, Daniel Franco, Lucio García, Carlos Zapata

**Affiliations:** 1 Department of Zoology, Genetics and Physical Anthropology, University of Santiago de Compostela, 15782 Santiago de Compostela, Spain; javier.bernal.pampin@gmail.com (J.B.); daniel.mouzo.calzadilla@usc.es (D.M.); mariadolores.lopez@usc.es (M.L.-P.); 2 Meat Technology Center of Galicia, 32900 San Cibrao das Viñas, Ourense, Spain; danielfranco@ceteca.net (D.F.); luciogarcia@ceteca.net (L.G.)

**Keywords:** *Solanum tuberosum*, patatin, seed storage proteins, vegetative storage proteins, tuber phosphoproteome, targeted two-dimensional electrophoresis

## Abstract

The role of the protein phosphorylation mechanism in the mobilization of vegetative storage proteins (VSPs) is totally unknown. Patatin is the major VSP of the potato (*Solanum tuberosum* L.) tuber that encompasses multiple differentially phosphorylated isoforms. In this study, temporal changes in the phosphorylation status of patatin isoforms and their involvement in patatin mobilization are investigated using phosphoproteomic methods based on targeted two-dimensional electrophoresis (2-DE). High-resolution 2-DE profiles of patatin isoforms were obtained in four sequential tuber life cycle stages of Kennebec cultivar: endodormancy, bud break, sprouting and plant growth. In-gel multiplex identification of phosphorylated isoforms with Pro-Q Diamond phosphoprotein-specific stain revealed an increase in the number of phosphorylated isoforms after the tuber endodormancy stage. In addition, we found that the phosphorylation status of patatin isoforms significantly changed throughout the tuber life cycle (*P* < 0.05) using the chemical method of protein dephosphorylation with hydrogen fluoride-pyridine (HF-P) coupled to 2-DE. More specifically, patatin phosphorylation increased by 32% from endodormancy to the tuber sprouting stage and subsequently decreased together with patatin degradation. Patatin isoforms were not randomly mobilized because highly phosphorylated Kuras-isoforms were preferably degraded in comparison to less phosphorylated non-Kuras isoforms. These results lead us to conclude that patatin is mobilized by a mechanism dependent on the phosphorylation status of specific isoforms.

## 1. Introduction

The molecular mechanisms controlling the mobilization of seed storage proteins (SSPs) play a critical role in plant reproduction [1,2]. Storage proteins accumulate progressively during seed development providing the nutrients necessary for germination and plant growth [3,4,5]. Phosphoproteomic studies have shown that SSPs (i.e., albumins, globulins, prolamines and glutelins) are abundantly phosphorylated and thereby phosphorylation may play a key role in the accumulation of storage proteins during seed development and mobilization during germination [6,7,8]. Studies in the model plant *Arabidopsis thaliana* suggest that a complex crosstalk of protein kinases, phosphatases and phytohormones are mainly involved in the regulatory networks modulating the phosphorylation status of SSPs [9,10,11,12]. An added complexity is that SSPs often exhibit multiple differentially phosphorylated isoforms with contrasting temporal patterns during seed development and germination [6,8,13,14,15,16]. The biological meaning of these isoform-dependent temporal phosphorylation patterns is not well understood. Lopez-Pedrouso et al. [17] reported that highly phosphorylated isoforms of the phaseolin in common beans were preferentially degraded in dry-to-germinating seed transition, suggesting a phosphorylation-dependent mobilization mechanism.

VSPs are a particular type of differentiated and less studied plant storage proteins located in vegetative tissues [1,3,18]. Patatin is the most abundant VSP in the potato (*S*. *tuberosum*) tuber, accounting for up to circa 45% of the total soluble protein [1,3,19]. Unlike most other plant storage proteins, patatin also exhibits different enzymatic activities including a non-specific lipid acyl hydrolase (LAH) activity with a probable role in plant defense mechanisms [1,20,21,22]. Phosphorylated isoforms of VSPs were identified for the first time in the patatin protein [23]. It is a very complex protein that comprises multiple differentially phosphorylated isoforms within—and between—potato cultivars [23,24]. A 2-DE-based phosphoproteomic study enabled the identification of 20 differentially phosphorylated patatin isoforms in Kennebec cultivar, with phosphorylation rates (*PR*s) varying between 4.6 and 52.3% [23]. This finding raises the question of whether VSPs are mobilized by a phosphorylation-dependent mechanism as occurs in the common bean. It is noteworthy that the germination of dry after-ripening seeds can be easily activated in the laboratory with water imbibition to assess changes in the phosphorylation status of SSPs in dry-to-germinating seed transition. However, this is a more difficult issue to solve experimentally in potatoes because tubers undergo continuous growth between endodormancy and sprouting stages. The endodormancy phase ends with the construction of phloem structures that allow for the provision of nutrients to the apical meristem to produce the first 2-mm-long apical bud (bud break) and then tuber progresses until the sprouting phase [5,25]. Accordingly, changes in the status of phosphorylation associated with the onset of patatin mobilization can potentially occur within the extensive time period comprised between the endodormancy and sprouting stages.

**Figure 1:**
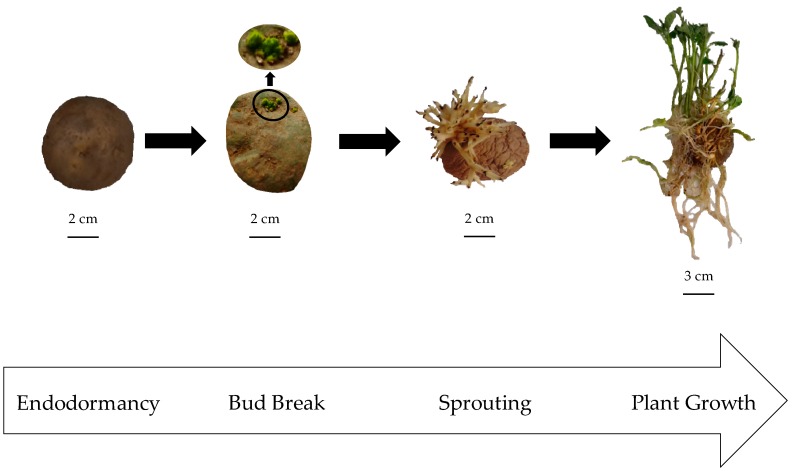
Four sequential stages of the potato tuber life cycle used to assess changing status of patatin phosphorylation in Kennebec cultivar: endodormancy, bud break (2 mm apical bud length), sprouting (5 cm bud length) and plant growth (12 cm stem length).

This study aimed to assess whether the existence of differentially phosphorylated patatin isoforms in potato tubers is related to the mechanism of patatin mobilization. For this purpose, a comprehensive analysis of temporal phosphorylation changes in patatin was performed in tubers of Kennebec cultivar (Figure 1). We used targeted 2-DE-based proteomic methods enabling the separation of multiple storage protein isoforms with high resolution and reproducibility as well as the efficient in-gel quantitation of their phosphorylation levels [8,17,23,24,26,27]. The results will contribute to understanding the mechanisms underlying the mobilization of VSPs.

## 2. Results and Discussion

### 2.1. Reference Patatin Profiles throughout the Tuber Life Cycle

**Figure 2:**
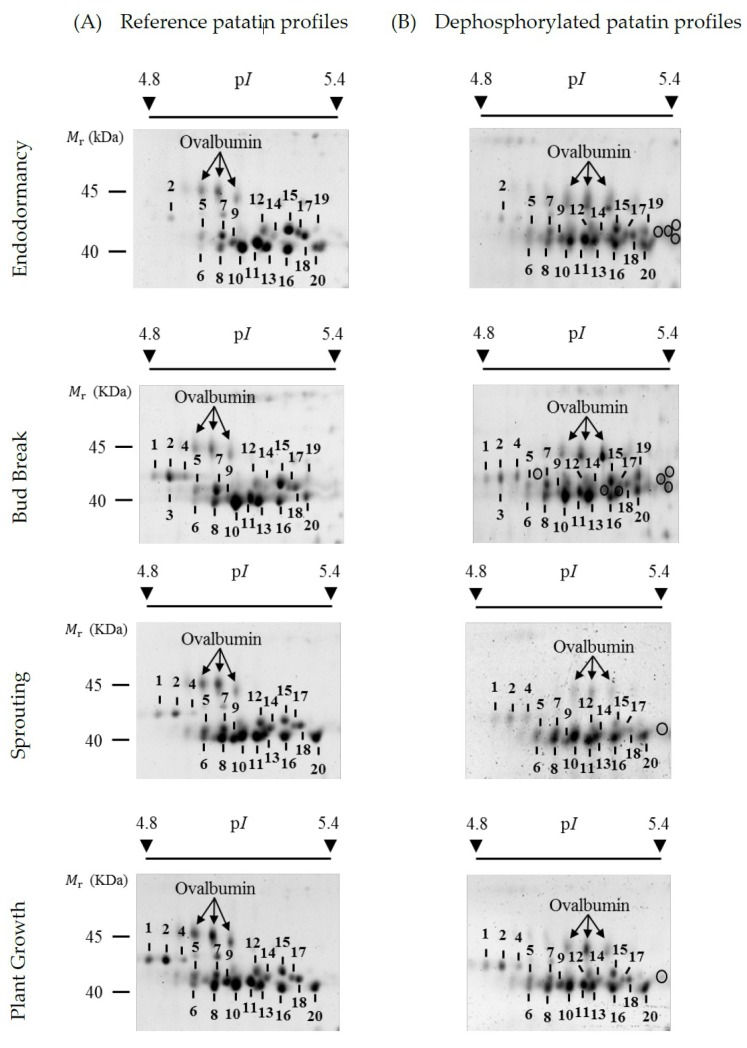
Representative two-dimensional electrophoresis (2-DE) gel images of reference and dephosphorylated profiles of patatin at four different tuber life stages of the Kennebec cultivar: endodormancy, bud break, sprouting and plant growth. (**A**) Reference profiles on 2-DE gels stained with Pro-Q Diamond and post-stained with SYPRO Ruby fluorescent dyes. Phosphorylated patatin spots are numbered. (**B**) Dephosphorylated patatin profiles on 2-DE gels stained with SYPRO Ruby from total tuber protein extracts treated with hydrogen fluoride-pyridine (HF-P). Circles show the gel position of newly arisen patatin spots following chemical dephosphorylation with HF-P. The arrows indicate the gel position of ovalbumin (45.0 kDa) phosphoprotein marker.

Figure 2A shows representative high-resolution profiles of patatin on 2-DE gels stained with SYPRO Ruby total-protein stain at each of the four potato life cycle stages of the Kennebec cultivar analyzed: endodormancy, bud break, sprouting and plant growth. The relative molecular mass (*M*_r_) of 2-DE patatin spots ranged from 40.0 to 45.0 kDa and their isoelectric points (p*I*s) ranged from 4.8 to 5.4 as previously reported in bud break stage of the same cultivar [23]. Qualitative variations in the number of patatin spots were detected throughout tuber stages. Most variations were identified after endodormancy stage on more acidic gel positions involving spot Numbers 1–4. Thus, spot Numbers 1, 2 and 4 were identified in bud-break, sprouting and plant growth stages, while only spot Number 2 was identified in the endodormancy phase.

Previous protein identification of 2-DE patatin spots in the bud break tuber stage of the Kennebec cultivar using matrix-assisted laser desorption/ionization time-of-flight (MALDI-TOF) and MALDI-TOF/TOF mass spectrometry (MS) revealed that spot Numbers 1, 2 and 4 contained the Patatin-3 Kuras 1 (PT3K1) isoform, while the other spots (i.e., spot Numbers 5–20) contained non-Kuras patatin isoforms [23]. The PT3K1 isoform was unequivocally identified because it is the patatin isoform with the most highly differentiated sequence [28]. In the present study, we confirmed these previous identifications by MALDI-TOF and MALDI-TOF/TOF MS from 2-DE gels in the endodormancy phase (Supplementary Tables S1 and S2; Supplementary Materials). Therefore, MS analysis leads us to conclude that Kuras isoforms explain most variations in the number of patatin spots throughout the tuber stages.

### 2.2. Changes in Patatin Abundance in the Tuber Life Cycle

**Figure 3:**
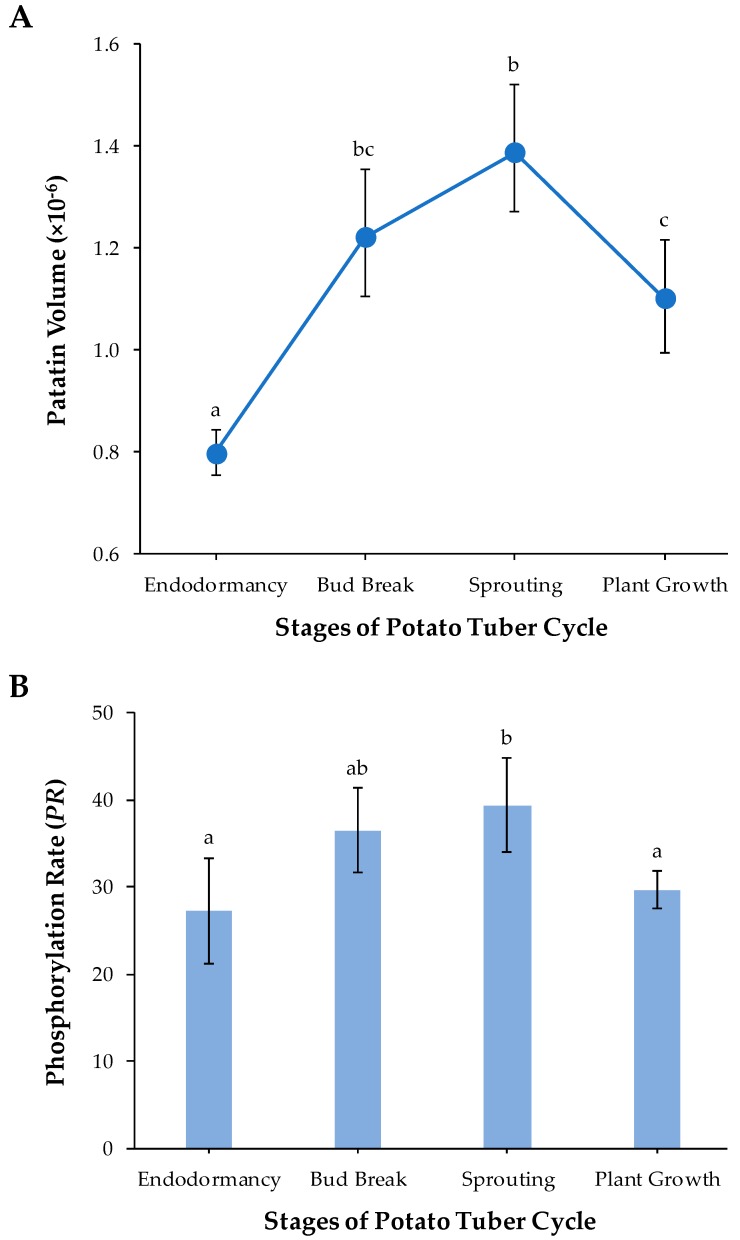
(**A**) Dynamic changes in the patatin abundance through the different stages of the potato tuber life cycle: endodormancy, bud break, sprouting and plant growth. In each tuber stage, mean patatin quantity over four biological replicates is represented together with their Bonferroni-corrected 95% bootstrap CIs. (**B**) Mean *PR* values together with their 95% confidence interval obtained from four biological replicates in endodormancy, bud break, sprouting and plant growth potato tuber stages of the Kennebec cultivar. Different lower case letters (a–c) indicate statistically significant differences (*p* < 0.05) between sample groups by the post hoc Tukey-Kramer test following the one-way analysis of variance (ANOVA) test.

A rough estimation of the amount of patatin in each of the four tuber life cycle stages was obtained by means of the sum of the patatin spot volumes assessed by PDQuest software. Mean values of the amount of protein along with the bias-corrected 95% bootstrap confidence intervals, adjusted by Bonferroni’s correction for multiple comparisons, were obtained in each potato stage from four biological replicate gels. Figure 3A shows that patatin abundance significantly increased from endodormancy to sprouting (*p*-value < 0.05) and then decreased to plant growth potato stage (*p* < 0.05). These results have a clear biological significance. Patatin achieved maximum abundance at the time of tuber sprouting when storage protein reserves are necessarily broken down into seedling growth. In agreement with these results, Lehesranta et al. [29] previously reported from 2-DE data that most patatin isoforms in the Desiree cultivar increased during tuber development achieving high amounts at the onset of sprouting and subsequently decreased in fully sprouted tubers. Quantification of patatin from Ranger Russet cultivar by LAH activity using p-nitrophenyl myristate as substrate showed its pronounced decrease during plant establishment [30]. In addition, analyses of the transcriptome throughout the potato tuber life cycle are in accordance with those proteomic studies. Thus, a comparative analysis of transcripts encoding patatin throughout the potato (Kennebec cultivar) tuber cycle based on cDNA-AFLP fingerprinting (Bintje cultivar) and expressed sequence tag (EST) libraries have shown that patatin transcripts were relatively higher at the onset of tuber sprouting [31,32]. Chemically (bromoethane) induced cessation of dormancy coupled to microarrays constructed from potato EST libraries has also shown a decrease in transcripts that encode for patatin after the onset of sprouting in tubers of the Russet Burbank cultivar [33]. Temporal studies of patatin concentration, activities of proteases and protease inhibitors suggest correlative changes during tuber development and plant establishment. There is increasing evidence that protease inhibitors (cys-protease inhibitor potato multicystatin, the Kunitz protease inhibitor family and others) may facilitate patatin accumulation in developing tubers by attenuating the activity of proteases, while catabolism of protease inhibitors may facilitate patatin mobilization following tuber sprouting [30,33,34,35].

### 2.3. Identification of Phosphorylated Patatin Isoforms along the Tuber Life Cycle

Patatin profiles on 2-DE gels stained with Pro-Q Diamond phosphoprotein-specific fluorescent dye in endodormancy, bud break, sprouting and plant growth tuber stages of Kennebec cultivar are shown in Supplementary Figure S1 (Supplementary Materials). All 2-DE patatin spots contained phosphorylated isoforms because they were reproducibly Pro-Q-Diamond-stained across biological replicates. These results suggest that the above-mentioned variations in the number of Kuras isoforms contribute to the increase of patatin phosphorylation after the endodormancy stage.

### 2.4. Changes in Patatin Phosphorylation Status during Tuber Life Cycle

The phosphorylation level of patatin spots was assessed using the method of chemical dephosphorylation with hydrogen fluoride-pyridine (HF-P). 2-DE patatin profiles obtained from total tuber (Kennebec cultivar) protein extracts dephosphorylated with HF-P in the tuber phases analyzed are shown in Figure 2B. First of all, HF-P was a very effective treatment of protein dephosphorylation as previously shown [17,23]. Note that the isoforms of the ovalbumin phosphoprotein marker completely shifted to more basic gel positions after HF-P treatment, as a result of the loss of phosphate groups.

The phosphorylation level of each patatin spot was estimated using the *PR* coefficient. This coefficient calculates the percentage by which the total spot volume (untreated protein samples) decreased after protein dephosphorylation with HF-P (treated protein samples). Therefore, it is a phosphorylation measure independent of the existing variations in the amount of patatin over spots. Mean (± SE) *PR* values of patatin spots obtained from four biological replicate gels in the distinct sample groups are shown in Table 1. It can be seen that all spots contained phosphorylated isoforms because *PR*-values were always higher than zero, which is in agreement with those results obtained with Pro-Q Diamond stain. In addition, *PR*-values exhibited remarkable differences among spots when mean values ranged from 10.9 (spot Number 11/endodormancy) to 53.2% (spot Number 14/sprouting). No *PR*-value achieved the maximum value of 100%, which indicates that each patatin spot contained a variable mixture of phosphorylated and unphosphorylated isoforms.

Parametric statistical tests were used to test for differences in *PR* over the different potato tuber phases because their sampling distributions did not significantly deviate from the theoretical normal distribution (*p* > 0.05; Shapiro-Wilk test). The one-way analysis of variance (ANOVA) test revealed that mean values of *PR* differed significantly (*p* < 0.01) among sample groups. More specifically, we found by using the Tukey-Kramer method for multiple comparisons that *PR*-values increased by 32% from endordormacy release to tuber sprouting (*p* < 0.05) and subsequently decreased up to the plant-growth stage (*p* < 0.05), achieving levels close to the endodormancy stage (Figure 3B). Concomitant temporal changes of the amount of patatin (Figure 3A) and phosphorylation levels (Figure 3B) suggest that protein phosphorylation is involved in the regulation of the synthesis and mobilization of patatin. A targeted 2-DE-based study in common bean also showed that the phosphorylation status of phaseolin isoforms increased in dry seed-to-gemination transition [17].

The advantage of targeted 2-DE is that the global phosphorylation of storage proteins can be estimated more accurately by considering the complete set of isoforms. In contrast, non-targeted 2-DE only enables the analysis of a limited number of storage protein isoforms. A number of non-targeted 2-DE proteomic studies have reported temporal phosphorylation changes in SSPs during seed development and germination. In white lupin, only vicilin family proteins were phosphorylated during seed development, both vicilins and legumins were phosphorylated in mature seeds and only vicilins increased their phosphorylation level at 2 d after the onset of germination [13]. In rice, a phosphoproteomic study in different phases of seed germination revealed that storage proteins achieved the highest levels of phosphorylation at the same time as their degradation [15]. In wheat, increased phosphorylation levels of globulin 3 were detected at 12 h following imbibition [16]. In Helianthus, 11S globulins showed increased levels of phosphorylation following completation of germination [14]. In oilseed rape, the amount of phosphorylated cruciferin subunits exhibited contrasting trends over distinct sequential phases of seed development, either increasing or decreasing their phosphorylation level in the late maturation stage [6]. Unfortunately, the global phosphorylation trend of the subunits identified was not reported. Overall, the join analysis of targeted and non-targeted 2-DE studies on temporal phosphorylation changes in SSPs and patatin VSP suggests that storage proteins tend to increase their phosphorylation levels during seed/tuber development until immediately after the onset of the seed germination/tuber sprouting phase.

A reduced number of studies have shown that phytohormones, protein kinases and protein phosphatases cooperate in largely unknown complex signaling networks that specifically regulate the changing status of storage protein phosphorylation. Seed phosphoproteomic studies in Arabidopsis indicate a key regulatory role of phytohormone abscisic acid (ABA). It has been suggested that cruciferin tyrosine-phosphorylation is modulated by ABA, which might help to prevent cruciferin proteolysis [11]. The ABA-insensitive gene (*abi1*) encodes a protein phosphatase that is a negative regulator of ABA-dependent kinase signaling during seed development and interacts with the ubiquitin-proteosome system [10,36,37]. Wan et al. [10] reported an increased phosphorylation level of the seed storage protein cruciferin in *abi1-1* mutant seeds.

**Figure 4:**
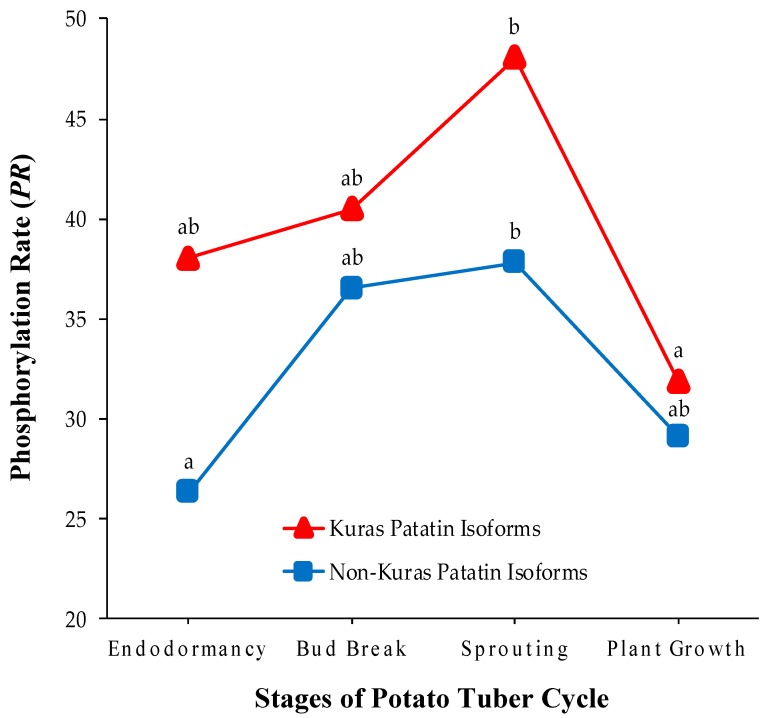
Temporal changes in the phosphorylation status of Kuras and non-Kuras patatin isoforms in the different stages of the potato tuber life cycle: endodormancy, bud break, sprouting and plant growth. Values on the y-axis are means of *PR* computed from four biological replicate gels over Kuras (triangles) and non-Kuras (squares) patatin spots. Different lower case letters (a and b) indicate statistically significant differences (*p* < 0.05) between sample groups by the post hoc Tukey-Kramer test following the one-way ANOVA test.

Another important issue is to elucidate why the phosphorylation status decreases when patatin is degraded following tuber sprouting. Note that the phosphorylation status should remain unchanged as long as patatin isoforms are randomly degraded. The answer to this question can be found by analyzing isoform-dependent phosphorylation changes throughout the tuber life cycle. Mean *PR*-values for Kuras and non-Kuras isoforms in the four potato stages are graphically represented in Figure 4. It is noteworthy that *PR*-values were always higher in Kuras than in non-Kuras isoforms (*p* < 0.01, Wilcoxon’s signed-ranks test). Therefore, Kuras isoforms made a remarkable contribution, both qualitatively and quantitatively, to the variations of patatin phosphorylation in tuber stages. In addition, *PR*-values of Kuras and non-Kuras isoforms changed significantly in the potato tuber cycle (*p* < 0.05, one-way ANOVA test), following similar trends to those observed for all the isoforms. However, Kuras isoforms experienced a noticeable decrease in *PR* (33%) from sprouting to plant growth stage (*p* < 0.01), whereas no parallel decrease was detected in non-Kuras isoforms (*p* > 0.05). This finding supports the conclusion that differentially-phosphorylated patatin isoforms were not randomly degraded after tuber sprouting. It appears, therefore, that proteases predominantly selects highly phosphorylated isoforms during patatin mobilization. In agreement with these results, previous phosphoproteomic research in common bean showed that highly phosphorylated phaseolin isoforms are preferentially degraded at the onset of seed germination [17].

The challenge is how to explain the fact that highly phosphorylated isoforms were preferentially degraded by proteolytic enzymes. Wan et al. [10] suggested that the level of phosphorylation/dephosphorylation might destabilize tertiary and quaternary interactions of storage proteins leading to proteolytic cleavage. Patatin is an 88-kDa dimer, each subunit has approximately 366 aminoacids (17 Tyr) with negative and positive charges of the side-chains randomly distributed throughout the protein sequence, while amino acid residues are highly structured containing about 33% α-helical and 45% β-stranded structures [38,39]. It could be hypothesized that an excess of phosphates with a negative charge in highly phosphorylated patatin isoforms might disrupt the structure and facilitate proteolytic degradation. In silico prediction of patatin phosphopeptides/phosphosites from MS data suggests pronounced phosphopeptide/phosphosite variations between endodormancy and bud break tuber stages (Supplementary Table S3; Supplementary Materials). However, a more precise knowledge of the positions of phosphosites and stoichiometry throughout the tuber life cycle using phosphopeptides enrichment procedures coupled to MS analysis would be needed to explore phosphosite interactions with the tertiary and quaternary structure of the patatin. Unfortunately, this is not an easy task because it would require the isolation and purification of 20 highly homologous (frequently higher than 90%) and immunologically indistinguishable patatin isoforms [40,41,42] from tuber protein extracts prior to the application of enrichment procedures. To our knowledge, this wide diversity of patatin isoforms has only successfully been separated by targeted 2-DE to date. Pots et al. [42] reported that patatin isoforms pools (A, B, C, D) isolated from the Bintje variety showed no differences in conformation and thermal conformational stability before the sprouting of potatoes. In this regard, comparison of structural properties and stability of isoforms has not been reported in fully sprouted tubers when variably phosphorylated patatin isoforms were accumulated and differentially degraded. It would be necessary to perform a comparative analysis of structural properties and stability among all previously isolated patatin isoforms from protein extracts, following tuber sprouting, in order to achieve a reliable conclusion. An alternative explanation is that increased phosphorylation levels of patatin may induce its degradation by proteases. A number of studies have shown that reversible phosphorylation/dephosphorylation in plant proteins is a significant post-translational regulatory mechanism inducing degradation by the ubiquitin-proteosome pathway [15,43,44].

Taken together, our observations open up new ways of unraveling the complex regulatory mechanisms underlying not only phosphorylation-dependent patatin mobilization, but also to explore how patatin phosphorylation affects its enzymatic activities. It is noteworthy that patatin contains a conserved amino acid motif (Gly-X-Ser-X-Gly) at the position 75-79 of the sequence of patatin Pat17, which is a catalytic domain responsible for its LAH activity with an insecticidal function [45,46]. Mutational and enzymatic activity studies revealed that the Ser residue of the catalytic domain is critical for esterase and insecticidal activity [45,46,47]. Therefore, it would be interesting to assess whether the active site Ser residue is phosphorylated. In vitro assays with purified patatin suggest that activation of LAH activity might be caused through phosphorylation by a protein kinase [48,49]. No evidence for phosphorylation in this Ser residue was found in our prospective identification of patatin phosphosites (Supplementary Table S3; Supplementary Materials). However, the application of targeted MS analysis using high resolution technologies should be able to answer this important question.

Further follow-up studies are clearly needed to assess the potential effects of patatin phosphorylation on stability and LAH enzymatic activity, protease activity, as well as the regulatory mechanisms linked to changes in patatin phosphorylation status by the concerted action of kinases/phosphatases. In particular, the combined use of targeted and non-targeted 2-DE-based proteomics together with gel-free MS-driven proteomics could contribute very significantly to the understanding of the complex crosstalk between patatin phosphorylation and interacting proteins.

## 3. Materials and Methods

### 3.1. Plant Material

Potato (*S. tuberosum*) tubers of Kennebec cultivar were collected from an experimental field located at Xinzo de Limia (Orense, Spain). Four biological replicates were obtained in four tuber life cycle stages: endodormancy, bud break, sprouting and plant growth (Figure 1). Endodormant tubers were collected immediately after potato harvest, while bud-broken (2 mm apical bud length) and sprouting tubers (about 5 cm bud length) were obtained in the dark at room temperature. Potato plants (about 12 cm stem length) were grown in pots with fertilized soil and water supply inside a growth chamber with photoperiod (16/8 h light/dark) and temperature (22/18 °C light/dark) control. Four tubers from each tuber stage were eventually cut into small pieces, lyophilized after grinding the tissue into a fine powder with liquid nitrogen using a pre-cooled mortar and pestle, and stored separately at −80 °C before proteomic analysis. All experimental data in bud break stage were previously reported [17].

### 3.2. Tuber Protein Extraction

Total protein was extracted from lyophilized tubers using the phenol method based on phenol extraction coupled to ammonium acetate/methanol precipitation [50]. 200 mg of lyophilized sample was suspended in 3 mL of extraction buffer (500 mM Tris-HCl; 700 mM sucrose; 50 mM ethylenediaminetetraacetic acid, EDTA; 100 mM KCl; 1 mM phenylmethylsulfonyl fluoride, PMSF; 2% dithiothreitol, DTT; pH 8.0). An equal volume of phenol saturated with Tris-HCL (pH 6.6–7.9) was added. The sample was centrifuged for 15 min at 5000× *g* and 4 °C (Labofuge 400 R Heraeues, St Louis, MO, USA), and the phenolic phase was recovered. The precipitation solution (0.1 M ammonium acetate in cold methanol) was added, the pellet was washed (10 mM DTT and 80% acetone) and re-suspended in lysis buffer (7 M urea; 2 M thiourea; 4% CHAPS; 10 mM DTT; and 2% Pharmalyte^TM^ pH 3–10, GE Healthcare, Uppsala, Sweden). The protein concentration was quantified prior electrophoresis using a modified Bradford assay kit (CB-XTM Protein Assay kit; GBiosciences, St Louis, MO, USA) on a CroMate^®^ 4300 Microplate Reader. (Awareness Technology, Palm City, FL, USA) and bovine serum albumin (BSA) as standard.

### 3.3. Protein Chemical Dephosphorylation

Protein extracts from the four tuber stages were chemically dephosphorylated with hydrogen fluoride-pyridine (HF-P) as described previously [51], with some modifications [17,23]. Tuber protein extract (1 mg) was added to 250 μL of HF-P (70%) and incubated for 2 h in an ice water bath. The solution was neutralized with 10 M NaOH and desalinated by using Amicon Ultra-4 centrifugal filter devices (Millipore, Billerica, MA, USA) and then eluted in 300 μL of lysis buffer. Protein purification was eventually carried out using the Clean-Up kit (GE Healthcare, Chicago, IL, USA). The ovalbumin phosphoprotein marker (45.0 kDa, Molecular Probes, Leiden, the Netherlands) was used to control the efficiency of the dephosphorylation reaction. A quantitative comparison of patatin spot volumes on two-dimensional (2-DE) gels from treated versus untreated protein samples of the same tuber was used to estimate their levels of phosphorylation. Four independent biological replicates were used at each tuber stage (i.e., 16 tubers and 32 gels).

### 3.4. Two-Dimensional Electrophoresis (2-DE)

High-resolution patatin profiles from protein extracts were obtained using previously described targeted 2-DE protocols [23,24]. Briefly, first-dimension isoelectric focusing (IEF) was conducted on a PROTEAN^®^ IEF Cell (Bio-Rad Laboratories, Hercules, CA, USA) system using 24-cm-long immobilized pH gradient (IPG) strips (ReadyStrips™, Bio-Rad Laboratories) with linear pH gradient (pH 4–7). Protein extracts (75 µg) dissolved in lysis buffer were mixed with rehydration buffer (7 M urea, 2 M thiourea, 4% CHAPS, 0.002% bromophenol blue) to a final volume of 450 µL, 0.6% DTT and 1% IPG buffer (Bio-Rad Laboratories). The mixture was loaded onto initially rehydrated (50 V for 12 h) gel strips and then an increasing voltage was applied up to 70 kVh. Focused strips were subsequently equilibrated with two equilibration buffers (buffer I: 50 mM Tris pH 8.8, 6 M urea, 30% glycerol and 2% SDS; 1% DTT; buffer II: 50 mM Tris pH 8.8, 6 M urea, 30% glycerol and 2% SDS; 2.5% iodoacetamide) for 15 min each at room temperature. For second-dimension separation, strips were transferred to 10% sodium dodecyl sulphate-polyacrylamide gel electrophoresis (SDS-PAGE) gels (24 × 20 cm) and run on an Ettan™ DALTsix multigel electrophoresis system (GE, Healthcare, Uppsala, Sweden) at 16 mA/gel for 15 h. Molecular-mass markers ranging from 15 to 200 kDa (Fermentas, Burlington, Ontario, Canada) were loaded into a lateral well of SDS-PAGE gels.

### 3.5. Gel Staining for Total Protein and Phosphoproteins

Gel staining for total patatin was performed with SYPRO Ruby fluorescent dye (Lonza, Rockland, ME, USA) following the manufacturer’s instructions. In-gel identification of phosphorylated patatin isoforms was conducted with Pro-Q Diamond phosphoprotein-specific stain (Molecular Probes) following the protocol described in Agrawal and Thelen [52], with some minor modification [17]. The PeppermintStick™ (Molecular Probes, Thermo Fisher Scientific, Waltham, MA, USA) phosphorylated and unphosphorylated molecular weight markers were added to tuber protein extract prior 2-DE as positive and negative controls of Pro-Q Diamond staining. Gels stained with Pro-Q Diamond were subsequently stained with SYPRO Ruby to assess which of the patatin isoforms were phosphorylated on the same 2-DE gel.

### 3.6. Image Analysis

Images of 2-DE gels stained with SYPRO Ruby and Pro-Q Diamond fluorescent dyes were digitalized with the Gel Doc^TM^ XR + System (Bio-Rad Laboratories) and processed with the PDQuest^TM^ Advanced software v. 8.0.1 (Bio-Rad Laboratories). Automatic spot matching across gels following background subtraction and normalization with the total density of valid spots was checked manually. Only patatin spots reproducibly detected in at least three replicates were selected for further analyses. The experimental relative molecular mass (*M*_r_) and isoelectric point (p*I*) of spots was estimated from their gel position relative to the standard molecular mass markers and IPG-strip linear range of pH 4–7, respectively.

### 3.7. Mass Spectrometry (MS)

Kuras and non-Kuras patatin isoforms were identified at endodormancy tuber stage by MALDI-TOF and MALDI-TOF/TOF MS as previously [23]. Briefly, selected spots were subjected to in-gel proteolytic digestion with modified porcine trypsin (Promega, Madison, WI, USA) according to Jensen et al. [53]. Eluted tryptic peptides were dried in a SpeedVac (Thermo Fisher Scientific, Waltham, MA, USA) and stored at −20 °C. Dried peptide samples were redissolved with 0.5% formic acid and mixed with matrix solution (3 mg/mL CHCA in 50% ACN, 0.1% TFA). Peptide solution was deposited onto a 384 Opti-TOF MALDI target plate (Applied Biosystems, Foster City, CA. USA) using the “thin layer” procedure [54]. Mass spectra of each sample were adquired in the positive-ion reflector mode on a 4800 MALDI-TOF/TOF mass spectrometer (Applied Biosystems), Nd:YAG laser at 355 nm, an average accumulation of 1000 laser shots and at least three trypsin autolysis fragments for internal mass calibration. Tandem MS data were obtained from selected precursor ions with a relative resolution of 300 full width at half-maximum (FWHM) and metastable suppression. Automated analysis of peptide mass fingerprinting (PMF) and peptide fragmentation spectra was performed using the 4000 Series Explorer Software v. 3.5 (Applied Biosystems). GPS Explorer Software v. 3.6 using Mascot software v. 2.1 (Matrix Science, Boston, MA, USA) was used for the combined search of PMF and MSMS spectrum data against the *S. tuberosum* UniProtKB/Swiss-Prot databases. Database searching settings were precursor mass tolerance of 30 ppm, fragment mass tolerance of 0.35 Da, one missed cleavage site allowed, carbamidomethyl cysteine (CAM) as fixed modification and oxidized methionine as variable modification. All identifications and spectra were checked manually. The identification of Kuras and non-Kuras patatin isoforms required at the least three matched peptides and statistically significant (*p*-value < 0.05) Mascot probability scores. Prediction of patatin phosphopeptides/phosphosites was performed from spectra data allowing phosphoserine (PhosphoS), phosphotyrosine (PhosphoY) and phosphothreonine (PhosphoT) residues as variable modification to search against the UniProtKB/Swiss-Prot databases.

### 3.8. Statistical Analysis

The phosphorylation rate for each protein spot was quantified by the coefficient *PR* = [(X − Y)/X] × 100, in which X and Y are the 2-DE spot volumes from untreated and treated tuber protein extracts with HF-P, respectively [17]. Non-parametric bootstrap confidence intervals obtained by the bias-corrected percentile method were used to test changes in patatin abundance (mean values) during tuber life cycle stages, following the procedure described previously [55]. Bootstrap confidence intervals (95%) were adjusted with the Bonferroni correction for multiple comparisons. The usual statistical tests (i.e., Wilcoxon’s signed-ranks, Shapiro-Wilk, one-way ANOVA and the Tukey-Kramer post-hoc tests) were performed with the IBM SPSS Statistics 21 (SPSS, Chicago, IL, USA) software package.

## 4. Conclusions

In this study, we have provided, for the first time, confident evidence of qualitative and quantitative temporal changes in the phosphorylation status of patatin isoforms. It is also the first study on this subject in VSPs. Our data revealed that isoforms with major extent of phosphorylation accumulated progressively from endodormancy up to reaching the tuber sprouting stage. The joint analysis of previously published proteomic studies in distinct SSPs showed similar dynamic temporal patterns. These temporal patterns acquired their full meaning when our observations showed that highly phosphorylated isoforms were preferably degraded immediately after the onset of tuber sprouting. Similar results have been reported in the mobilization of the major common bean storage protein. Therefore, the evidence available suggests that degradation of storage proteins dependent on the status of phosphorylation of specific isoforms might be a general mobilization mechanism involving both VSPs and SSPs. Further research is clearly required to assess the signaling networks that trigger and regulate these complex processes.

## Figures and Tables

**Table 1:** Mean (± SE) phosphorylation rate (*PR*) of patatin spots in different stages of potato tuber (Kennebec cultivar) life cycle.

		*PR*				
Spot Number ^a^	p*I*	Endodormancy	Bud Break	Sprouting	Plant Growth	Mean (± SE)
1	4.84	N/A	39.8 ± 2.5	51.9 ± 5.2	33.1 ± 5.6	41.6 ± 5.5
2	4.88	38.1 ± 5.3	41.2 ± 6.1	52.4 ± 5.9	39.6 ± 5.7	42.8 ± 3.3
3	4.90	N/A	43.1 ± 2.9	N/A	N/A	N/A
4	4.93	N/A	N/A	39.7 ± 5.4	23.0 ± 4.6	31.4 ± 8.4
5	4.96	27.1 ± 1.9	42.8 ± 2.0	43.2 ± 6.4	30.5 ± 4.4	35.9 ± 4.2
6	4.96	N/A	52.3 ± 4.1	38.4 ± 4.4	27.5 ± 5.2	39.4 ± 7.2
7	5.02	32.3 ± 4.5	39.0 ± 4.1	42.5 ± 5.9	26.9 ± 5.8	35.2 ± 3.5
8	5.02	15.3 ± 3.8	27.2 ± 5.6	27.0 ± 4.6	29.1 ± 5.1	24.6 ± 3.2
9	5.05	27.3 ± 2.4	32.5 ± 3.3	40.5 ± 5.1	31.4 ± 5.7	32.9 ± 2.8
10	5.12	32.0 ± 4.0	30.5 ± 4.7	23.5 ± 6.1	40.0 ± 6.4	31.5 ± 3.4
11	5.12	10.9 ± 6.9	25.1 ± 4.3	26.4 ± 4.4	25.2 ± 6.3	21.9 ± 3.7
12	5.13	28.3 ± 2.4	51.4 ± 5.5	52.7 ± 4.2	31.9 ± 7.0	41.1 ± 6.4
13	5.14	N/A	N/A	N/A	26.0 ± 7.2	N/A
14	5.16	50.1 ± 4.9	44.3 ± 9.4	53.2 ± 7.5	33.3 ± 5.9	45.2 ± 4.4
15	5.20	41.4 ± 2.4	41.9 ± 3.8	44.1 ± 5.5	28.0 ± 5.3	38.8 ± 3.7
16	5.20	12.8 ± 3.1	27.0 ± 8.4	17.2 ± 4.3	25.1 ± 5.1	20.6 ± 3.3
17	5.23	23.8 ± 3.6	36.0 ± 2.4	39.8 ± 6.3	29.3 ± 4.6	32.2 ± 3.5
18	5.25	29.6 ± 5.3	34.2 ± 4.1	51.6 ± 4.5	22.9 ± 4.6	34.5 ± 6.1
19	5.29	N/A	N/A	N/A	N/A	N/A
20	5.27	11.6 ± 2.0	11.9 ± 4.6	25.6 ± 6.3	29.2 ± 4.9	19.6 ± 4.6
Mean (± SE)		27.2 ± 3.1	36.5 ± 2.5	39.4 ± 2.8	29.6 ± 1.1	

^a^ Gel position of numbered spots is shown in Figure 2. N/A = not applicable, absent spot in untreated and treaded protein samples or with volume below the limit of detection.

## Supplementary Materials

Supplementary materials can be found at https://www.mdpi.com/1422-0067/20/8/1889/s1.

## Abbreviations

2-DE	Two-dimensional electrophoresis
ABA	Abscisic acid
HF-P	Hydrogen fluoride-pyridine
LAH	Lipid acyl hydrolase
*M*_r_	Relative molecular mass
MALDI-TOF	Matrix-assisted laser desorption/ionization time-of-flight
MS	Mass spectrometry
p*I*	Isoelectric point
*PR*	Phosphorylation rate
SSP	Seed storage protein
VSP	Vegetative storage protein
