# Supplementary material for: The Major Storage Protein in Potato Tuber Is Mobilized by a Mechanism Dependent on Its Phosphorylation Status
Source: Int J Mol Sci. 2019 Apr 17;20(8):1889. doi: 10.3390/ijms20081889 (PMC6514604; doi:10.3390/ijms20081889)
Supplement: Supplementary file 1 [file ijms-20-01889-s001.pdf]

Supplementary Material

International Journal of Molecular Sciences

Supplementary Tables 1-3

Supplementary Figure 1

Table S1. Identification of Kuras and non-Kuras patatin isoforms along 2-DE gel spots in endodormant potato tubers of Kennebec cultivar by MALDI-TOF and MALDI-TOF/TOF MS.

Spot No.[a]	p*I* [b]	Patatin isoform[c]	Mascot score[c]	Sequence Coverage (%)[e]	Matched peptides[f]
2	4.88	Patatin-3-Kuras 1 (PT3K1)	571	58	13
5	4.96	Non-Kuras isoform	106	8	3
6	4.96	–	–	–	–
7	5.02	Non-Kuras isoform	200	40	11
8	5.02	Non-Kuras isoform	299	25	6
9	5.05	Non-Kuras isoform	324	28	7
10	5.08	Non-Kuras isoform	135	28	10
11	5.12	Non-Kuras isoform	337	32	9
12	5.13	Non-Kuras isoform	127	19	10
13	5.14	Non-Kuras isoform	178	26	10
14	5.16	Non-Kuras isoform	267	31	9
15	5.20	Non-Kuras isoform	146	17	7
16	5.20	Non-Kuras isoform	294	27	12
17	5.23	Non-Kuras isoform	181	20	8
18	5.25	Non-Kuras isoform	251	34	13
19	5.29	–	–		–
20	5.27	Non-Kuras isoform	215	27	10

[a]Gel position of numbered spots is shown in Figure 2. [b]Experimental p*I* value on 2-DE gels. [c]All identified proteins were matched to the patatin protein of *S. tuberosum*. [d]Statistically significant Mascot scores (*P*-value < 0.05). [e]Percentage of coverage of the entire amino acid sequence by matched peptides. [f]Total number of identified peptides matched for the patatin protein.

Table S2. Identification of the patatin isoforms along the 2-DE spot patterns in potato tubers of Kennebec cultivar in the endodormancy tuber stage by MALDI-TOF and MALDI-TOF/TOF MS.

Spot No.[a]	Protein identity (abbrev.)[b]	Accession	Mascot Score[c]	Match/Cov (%)[d]	Peptides identified	
					[M+H]+	Sequence
2	Patatin-3-Kuras 1 (PT3K1)	PT3K1_SOLTU	571	13/58	773.39	TPELDAK
					1,047.60	TNKPVIFTK
					1,167.60	YLMQVLQEK
					1,289.67	LAQEDPAFASIK
					1,448.72	DIIPFYFDHGPK
					1,499.78	IFEPSGFHLVEPK
					1,720.80	SVSEDNHETYEVALK
					1,757.93	VHQALTEVAISSFDIK
					1,825.91	QMLLLSLGTGTNSEFAK
					1,864.87	WGILQWMSPLWEMR
					3,137.41	SAASSYMNDYYLSTVFQALDSQNNYLR
					3,475.54	VQENALTGTATTFDDASVANMILLVQVGENLLK
					3,612.79	LADYFDVIGGTGTGGLLTAMITTPNENNRPFAAAK
5	Patatin-08	PAT08_SOLTU	106	3/8	876.42	DPAFASIR
	Patatin-D2	PATD2_SOLTU			1,013.49	TYTAEETAK
	Patatin-D3	PATD3_SOLTU			1,709.79	YLMQVLQENLGETR

6	–	–			–	–
7	Patatin-08	PAT08_SOLTU	200	11/40	759.36	SPELDAK
	Patatin-D2	PATD2_SOLTU			876.43	DPAFASIR
	Patatin-D3	PATD3_SOLTU			922.34	MDNNADAR
					1,013.49	TYTAEETAK
					1,047.58	TNKPVIFTK
					1,308.60	DNPETYEEALK
					1,709.82	YLMQVLQENLGETR
					1,728.83	MLLLSLGTGTTSEFDK
					1,757.91	VHQALTEVAISSFDIK
					2,882.16	MTDAASSYMTDYYLSTVFQAQNSQK
					3,569.59	VQENALTGTTTEMDDASEANMESLVQVGENLLK
8	Patatin-2-Kuras 1 (pt2k1)	PT2K1_SOLTU	299	6/25	1,031.49	HSQNNYLR
	Patatin-B1	PATB1_SOLTU			1,133.62	YLLQVLQEK
	Patatin-04	PAT04_SOLTU			1,305.65	LAQEDPAFSSIK
	Others	–			1,757.91	VHQALTEVAISSFDIK
					1,869.88	QMLLLSLGTGTNSEFDK
					3,642.78	LADYFDVIGGTSTGGLLTAMITTPNENNRPFAAAK
9	Patatin-2-Kuras 1 (pt2k1)	PT2K1_SOLTU	324	7/28	759.35	SPELDAK
	Patatin-B1	PATB1_SOLTU			1,031.49	HSQNNYLR
	Patatin-04	PAT04_SOLTU			1,133.64	YLLQVLQEK

	Others	–			1,305.65	LAQEDPAFSSIK
					1,757.90	VHQALTEVAISSFDIK
					1,869.88	QMLLLSLGTGTNSEFDK
					3,642.76	QMLLLSLGTGTNSEFDKAMITTPNENNRPFAAAK
10	Patatin-2-Kuras 1 (pt2k1)	PT2K1_SOLTU	135	10/28	758.38	SPELDAK
	Patatin-04	PAT04_SOLTU			948.52	FAKLLSDR
	Patatin-15	PAT15_SOLTU			1,030.49	HSQNNYLR
	Others	–			1,046.61	TNKPVIFTK
					1,062.43	TYTAEEAAK
					1,132.65	YLLQVLQEK
					1,304.66	LAQEDPAFSSIK
					1,436.68	DSPETYEEALKR
					1,756.94	VHQALTEVAISSFDIK
					1,868.92	QMLLLSLGTGTNSEFDK
11	Patatin group J1	PATJ1_SOLTU	337	9/32	922.36	MDNNADAR
	Patatin-04	PAT04_SOLTU			1,031.49	HSQNNYLR
	Patatin-06	PAT06_SOLTU			1,047.58	TNKPVIFTK
	Others	–			1,167.58	YLMQVLQEK
					1,305.65	LAQEDPAFSSIK
					1,757.92	VHQALTEVAISSFDIK
					1,853.90	QMLLLSLGTGTNSEFDK

					1,856.02	GIIPGTILEFLEGQLQK
					3,306.61	WMLAIQQMTNAASSYMTDYYISTVFQAR
12	Patatin-B2	PATB2_SOLTU	127	10/19	704.28	SLDYK
	Patatin-2-Kuras 1 (pt2k1)	PT2K1_SOLTU			706.28	ANKASH
	Patatin-04	PAT04_SOLTU			758.38	SPELDAK
	Others	–			1,030.49	HSQNNYLR
					1,046.61	TNKPVIFTK
					1,062.43	TYTAEEAAK
					1,132.65	YLLQVLQEK
					1,304.66	LAQEDPAFSSIK
					1,360.54	DSPETYEEALK
					1,436.68	DSPETYEEALKR
13	Patatin-2-Kuras 1 (pt2k1)	PT2K1_SOLTU	178	10/26	75.,38	SPELDAK
	Patatin-04	PAT04_SOLTU			1,030.49	HSQNNYLR
	Patatin-06	PAT06_SOLTU			1,046.61	TNKPVIFTK
	Others	–			1,062.43	TYTAEEAAK
					1,132.65	YLLQVLQEK
					1,304.66	LAQEDPAFSSIK
					1,360.54	DSPETYEEALK
					1,436.68	DSPETYEEALKR
					1,756.94	VHQALTEVAISSFDIK

					1,868.92	QMLLLSLGTGTNSEFDK
14	Patatin-03	PAT03_SOLTU	267	9/31	759.37	SPELDAK
	Patatin-05	PAT05_SOLTU			982.46	TYTAQEAAK
	Patatin-07	PAT07_SOLTU			1,031.49	HSQNNYLR
	Others	–			1,047.58	TNKPVIFTK
					1,133.64	YLLQVLQEK
					1,305.64	LAQEDPAFSSIK
					1,757.88	VHQALTEVAISSFDIK
					1,869.87	QMLLLSLGTGTNSEFDK
					3,642.78	LADYFDVIGGTSTGGLLTAMITTPNENNRPFAAAK
15	Patatin-2-Kuras 1 (pt2k1)	PT2K1_SOLTU	146	7/17	758.38	SPELDAK
	Patatin-04	PAT04_SOLTU			1,030.49	HSQNNYLR
	Patatin-B2	PATB2_SOLTU			1,046.61	TNKPVIFTK
	Others	–			1,062.55	TYTAEEAAK
					1,132.65	YLLQVLQEK
					1,304.66	LAQEDPAFSSIK
					1,436.68	DSPETYEEALKR
16	Patatin-B1	PATB1_SOLTU	294	12/27	758.38	SPELDAK
	Patatin-06	PAT06_SOLTU			947.56	FAKLLSNR
	Patatin-2-Kuras 1 (pt2k1)	PT2K1_SOLTU			1,030.49	HSQNNYLR
	Others				1,046.61	TNKPVIFTK

		–			1,062.43	TYTAEEAAK
					1,126.58	TNKPVIFTK
					1,132.65	YLLQVLQEK
					1,280.58	DSPETYEEALK
					1,304.66	LAQEDPAFSSIK
					1,436.68	DSPETYEEALKR
					1,756.94	VHQALTEVAISSFDIK
					1,868.92	QMLLLSLGTGTNSEFDK
17	Patatin-13	PAT13_SOLTU	181	8/20	758.44	SPELDAK
	Patatin-05	PAT05_SOLTU			921.36	MDNNADAR
	Patatin-03	PAT03_SOLTU			1,046.61	TNKPVIFTK
	Others	–			1,065.45	THTAQETAK
					1,204.57	AEEDPAFASIR
					1,261.65	VQENALTGTTTK
					1,360.67	RAEEDPAFASIR
					1,436.68	DSPETYEEALKR
18	Patatin-03	PAT03_SOLTU	251	13/34	758.38	SPELDAK
	Patatin-05	PAT05_SOLTU			921.36	MDNNADAR
	Patatin-07	PAT07_SOLTU			1,046.61	TNKPVIFTK
	Others				1,166.60	YLMQVLQEK
					1,204.57	AEEDPAFASIR

					1,261.65	VQENALTGTTTK
					1,360.67	RAEEDPAFASIR
					1,463.69	DNPETYEEALKR
					1,706.9	YLMQVLQEKLGETR
					1,756.94	VHQALTEVAISSFDIK
					1,789.74	YDGKYLMQVLQEK
					1,834.98	QLLLLSLGTGTNSEFDK
					2,274.15	ADDASEANMELLVQVGENLLK
19	–	–			–	–
20	Patatin-B1	PATB1_SOLTU	215	10/27	758.38	SPELDAK
	Patatin-06	PAT06_SOLTU			947.56	FAKLLSNR
	Patatin-2-Kuras 1 (pt2k1)	PT2K1_SOLTU			1,030.49	HSQNNYLR
	Others	–			1,046.61	TNKPVIFTK
					1,062.43	TYTAEEAAK
					1,132.65	YLLQVLQEK
					1,304.66	LAQEDPAFSSIK
					1,436.68	DSPETYEEALKR
					1,756.94	VHQALTEVAISSFDIK
					1,868.92	QMLLLSLGTGTNSEFDK

[a]Gel position of numbered spots is shown in Figure 2. [b]All identified proteins were matched to the patatin protein of *S. tuberosum*. [c]Statistically significant Mascot scores (*P*-value < 0.05). [d]Total number of identified spectra matched for the protein / Percentage of coverage of the entire amino acid sequence by matched peptides.

Table S3. Phosphorylated peptides and phosphorylation sites of the patatin in endodormancy and bud break tuber stages (Kennebec cultivar) identified by Mascot search from MALDI-TOF and MALDI-TOF/TOF MS data.

Spot no.[a]	Patatin name	Potato tuber stage	Phosphopeptides and phosphosites[b]	Position[c]
1	PT3K1	Bud Break	TPELDAK	162-168
2	PT3K1	Endodormancy	SVSEDNHETYEVALK	341-355
			WGILQWMSPLWEMR	266-279
		Bud Break	SVSEDNHETYEVALK	341-355
			WGILQWMSPLWEMR	266-279
9	Patatin mix	Bud Break	SFLILFFMILATTSSTCAK	6-24
10	Patatin mix	Endodormancy	TYTAEEAAK	269-277
		Bud Break	TYTAEEAAK	269-277
11	Patatin mix	Endodormancy	VHQALTEVAISSFDIK	144-159
		Bud Break	VHQALTEVAISSFDIK	144-159
			SFLILFFMILATTSSTCAK	6-24
			SFLILSVMILATTSSTFASLEEMVTVLSIDGGGIK	6-40
			MYDICYSAAAAPTYFPPHYFATNTINGDK	181-209
12	Patatin mix	Endodormancy	ANKASH	381-386
			SLDYK	247-251
			TYTAEEAAK	269-277
		Bud Break	TYTAEEAAK	269-277
13	Patatin mix	Endodormancy	TYTAEEAAK	269-277
		Bud Break	TYTAEEAAK	269-277
15	Patatin mix	Endodormancy	TYTAEEAAK	269-277
		Bud Break	TYTAEEAAK	269-277
			LEEMVTVLSIDGGGIK	25-44
			MYDICYSTAAAPIYFPPHHFVTHTSNGAR	180-208
			VQENALTGTTTEMDDA**S**EANMELLVQVGETLLK	319-351
16	Patatin mix	Endodormancy	TYTAEEAAK	269-277
		Bud Break	TYTAEEAAK	269-277
			SLDYK	247-251
			WMLAIQQMTNAASSYMTDYYISTVFQAR	283-310
			VQENALTGTTTEMDDA**S**EANMELLVQVGETLLK	319-351
17	Patatin mix	Endodormnacy	THTAEETAK	270-278
18	Patatin mix	Endodormancy	YDGKYLMQVLQEK	126-138
		Bud Break	THTAEETAK	270-278
			SLDYK	247-251
			SLNYK	248-252
			DSPETYEEALK	358-368
			VQENALTGTTTK	320-331
20	Patatin mix	Endodormancy	TYTAEEAAK	269-277
		Bud Break	TYTAEEAAK	269-277
			MATTK	1-5
			SLDYK	247-251
			WMLAIQQMTNAASSYMTDYYISTVFQAR	283-310

[a]Spot code numbers according to Fig. 2. [b]Phosphorylation sites are underlined. [C]Phosphopeptide position in the polypeptide sequence.

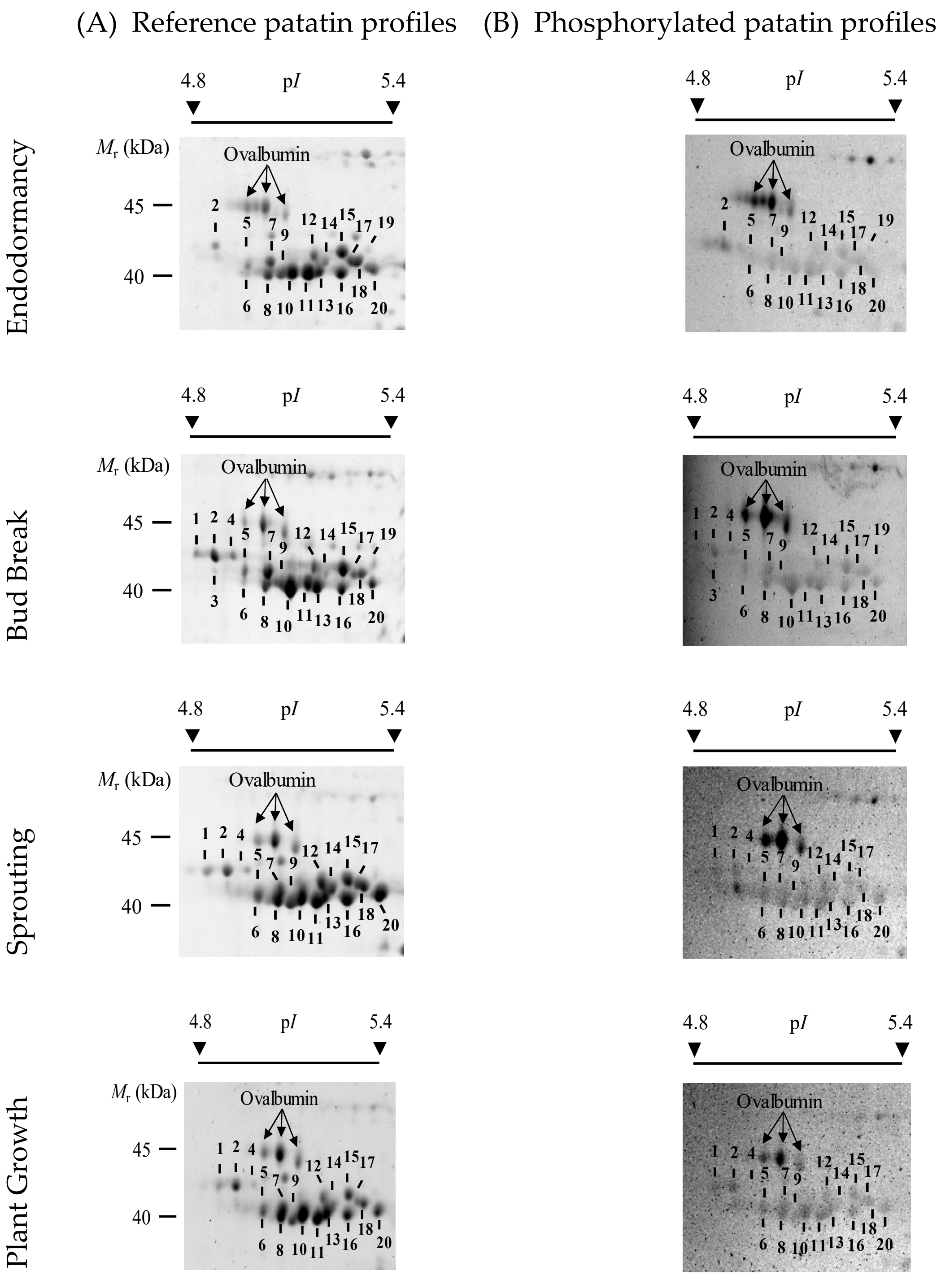

Figure S1. In-gel identification of phosphorylated patatin isoforms in Kennebec potato cultivar along different tuber life stages: endodormancy, tuber break, sprouting and plant growth. (**A**) Reference patatin profiles on 2-DE gels stained with SYPRO Ruby total-protein stain. (**B**) Phosphorylated patatin profiles on 2-DE gels stained with Pro-Q Diamond specific-phosphoprotein stain. Gel position of ovalbumin (45.0 kDa) phosphoprotein marker is shown.
